# Detection of *Listeria monocytogenes* in Food Using the Proofman-LMTIA Assay

**DOI:** 10.3390/molecules28145457

**Published:** 2023-07-17

**Authors:** Chunmei Song, Borui Wang, Yongzhen Wang, Jinxin Liu, Deguo Wang

**Affiliations:** 1 Key Laboratory of Biomarker Based Rapid-Detection Technology for Food Safety of Henan Province, Xuchang University, Xuchang 461000, China; 12016019@xcu.edu.cn (C.S.); 22009017@xcu.edu.cn (Y.W.); liujinxin@xcu.edu.cn (J.L.); 2 School of Food and Biological Engineering, Henan University of Science and Technology, Luoyang 471000, China; 200320090588@stu.haust.edu.cn

**Keywords:** Proofman probes, ladder-shape melting temperature isothermal amplification, *Listeria monocytogenes*, foodborne pathogens

## Abstract

Microbial factors, including bacteria, viruses, and other pathogens, are significant contributors to foodborne illnesses, posing serious food safety risks due to their potential for rapid growth and contamination. *Listeria monocytogenes* is one of the most common types of foodborne bacteria that can cause serious foodborne diseases or even fatalities. In this study, a novel nucleic acid amplification method called Proofman-LMTIA was employed to detect *Listeria monocytogenes* contamination in food. This method combines proofreading enzyme-mediated probe cleavage with ladder-shape melting temperature isothermal amplification. A positive recombinant plasmid was used as a control to ensure the accuracy of the detection results, and primers and Proofman probes were specifically designed for the LMTIA. Genomic DNA was extracted, the reaction temperature was optimized, and the primers’ specificity was verified using foodborne pathogens like Staphylococcus aureus, Escherichia coli O157:H7, and Salmonella. The sensitivity was assessed by testing serial dilutions of genomic DNA, and the method’s applicability was confirmed by detecting artificially contaminated fresh pork. The established LMTIA method exhibited both high specificity and sensitivity. At the optimal reaction temperature of 63 °C, the primers specifically identified *Listeria monocytogenes* contamination in pork at a concentration of 8.0 ± 0.7 colony-forming units (CFUs) per 25 g. Furthermore, the Proofman-LMTIA method was applied to test *Listeria monocytogenes* DNA in 30 food samples purchased from a Chinese retail market, and reassuringly, all results indicated no contamination. Proofman-LMTIA can serve as a reliable and rapid method for detecting *Listeria monocytogenes* in food, contributing to public health by safeguarding consumers from foodborne illnesses, and strengthening food safety regulations.

## 1. Introduction

Foodborne diseases, which are one of the most significant public health concerns worldwide, result in millions of reported cases annually in many countries [1]. *Listeria monocytogenes*, recognized as one of the four major foodborne pathogens, is a highly pathogenic bacterium [2]. During the analysis of microbial contamination in commercially available ready-to-eat foods in Shanghai from 2016 to 2020, *Listeria monocytogenes* was detected in over half of the sampled ready-to-eat food products. Among them, the highest detection rates were found in cold pot skewers at 6.27% (15/255), salads at 3.36% (17/506), Chinese-style cold mixed dishes at 2.71% (24/882), and cooked meat products at 2.69% (34/1263) [3]. *Listeria monocytogenes*, widely distributed in nature, exhibits stable physicochemical properties, and can still proliferate under environmental conditions ranging from 4 to 45 °C [4]. It is commonly found in food products, such as dairy and meat products, causing clinical symptoms, such as sepsis and meningitis, and in severe cases leading to fatalities [5,6]. Therefore, the effective detection of *Listeria monocytogenes* is of importance in preventing and controlling food safety incidents.

Currently, the commonly used detection methods for *Listeria monocytogenes* primarily include traditional culture-based techniques [7], immunological detection techniques [8,9,10], and biosensor-based detection techniques [11,12]. Traditional culture-based techniques have long detection cycles and are relatively complex to operate. Immunological techniques rely heavily on antibodies, which can limit their sensitivity. Biosensor-based techniques are constrained by higher cost regarding the short lifespan of electronic components, which restrict their application. Molecular biology techniques utilizing DNA, such as polymerase chain reaction (PCR) [13], loop-mediated isothermal amplification (LAMP) [14], droplet digital PCR [15], and real-time PCR [16,17], have proven to be highly effective in detecting *Listeria monocytogenes* in food. However, these methods often require expensive instruments and complex operations, resulting in long detection times. Therefore, there is an urgent need to develop a rapid, sensitive, and cost-effective method for the accurate detection of foodborne pathogens.

The ladder-shape melting temperature isothermal amplification (LMTIA) technique is a new nucleic acid isothermal amplification method developed by our team in 2021 [18]. LMTIA is an advanced method over the LAMP method, enabling stable nucleic acid amplification within a rapid timeframe (approximately 0.5 h), exhibiting the desired sensitivity and specificity, and producing reliable and accurate results. Compared to other nucleic acid amplification methods, in the LMTIA reaction, the generation of single-stranded templates is based on the difference in melting temperature (Tm) between the target sequence being amplified and its complementary strand, which does not rely on temperature variations or enzymatic activity. The feature of the DNA target will enhance the DNA synthesis efficiency to achieve robust and specific amplification of the target DNA. Additionally, the technique is easy to operate and presented with good consistency. LMTIA is currently applied for food adulteration [19,20,21] detection and virus detection [22], while its application in foodborne pathogen detection is still largely unexplored.

In this study, we aim to develop a rapid, sensitive, and specific detection method for *Listeria monocytogenes* by combining a novel probe, Proofman probe (proofreading enzyme-mediated probe cleavage) [23,24,25], with LMTIA technology. When using a Proofman probe, an additional DNA polymerase (Pfu) with proofreading activity was involved to cleave the probe from the 3′ end, producing a fluorescent signal. Proofman-LMTIA exhibits high specificity and detection accuracy because of its unique cutting and luminescence principles. Our research results contribute to the development and application of new technologies for the rapid detection of microbial contamination in food.

## 2. Results

### 2.1. Analysis of the Recombinant Plasmid Sequencing Results and Alignment with the Original Sequence

**Figure 1:**
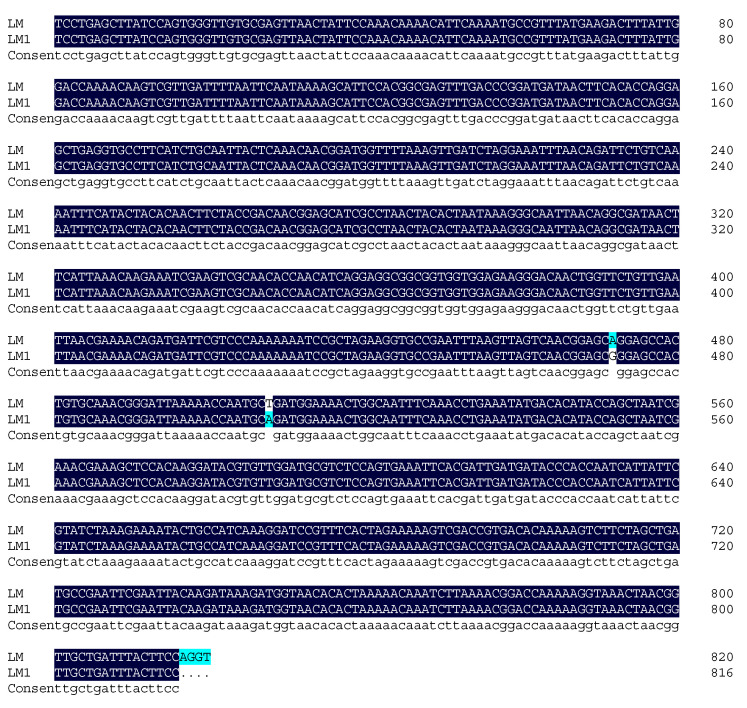
Comparison between the sequencing result of the recombinant plasmid and the original sequence.

The sequencing results of the recombinant positive plasmid were compared with the original sequence using DNAMAN v9.0 software. The alignment shown in Figure 1 showed high sequence homology of above 98%, confirming the successful construction of the recombinant plasmid.

### 2.2. Principle of the Proofman-LMTIA

**Figure 2:**
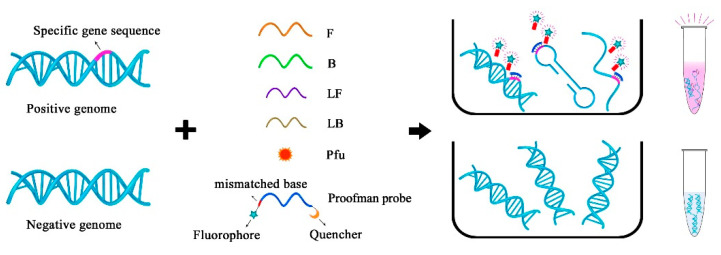
The schematic of the specific detection of LMTIA products using the Proofman probe.

The schematic strategy is shown in Figure 2, and the primers used for the LMTIA were designed according to the target sequence. The LMTIA can produce a single-chain structure, double-chain structure, and the dumbbell structure in the amplification phase. The Proofman probe, respectively, labeled with a fluorophore and quencher at the 3′ end and 5′ end, together with a thermostable proofreading DNA polymerase (Pfu), were introduced to realize the sequence-specific detection. Of note was that a deliberate mismatch at the 3′ end of the Proofman probe was necessary to trigger the 3′→5′ exonuclease activity of the proofreading enzyme Pfu. Upon binding of the Proofman probe to the target segment, the mismatched nucleotide would be cleaved by the Pfu, releasing the fluorophore. Since then, the cleaved probe can further serve as an extendable primer that enhances the isothermal amplification efficiency. The positive samples with the presence of the target gene can be amplified and detected by a pink fluorescence signal. Negative samples without carrying the target gene resulted in no amplification and the absence of a fluorescent signal. Based on the Proofman probe, the LMTIA amplification products can be detected specifically through the increase in the fluorescence intensity as the amplification reaction is proceeding. In our strategy, the sequence-specific detection of LMTIA products was achieved by the binding and cleavage of the Proofman probe, thus enhancing the accuracy of the LMTIA reaction.

### 2.3. Optimization of the Proofman-LMTIA Reaction Temperature

**Figure 3:**
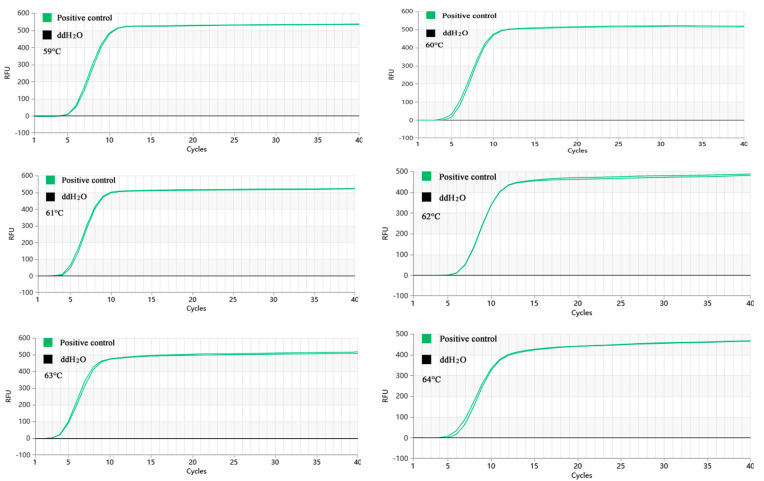
Proofman-LMTIA reaction at different temperatures.

As Figure 3 demonstrated, the Proofman-LMTIA reactions were conducted at temperatures of 59 °C, 60 °C, 61 °C, 62 °C, 63 °C, and 64 °C. The amplification was observed in the positive control, while no amplification was observed in the negative control (ddH_2_O as the template). The Proofman-LMTIA reaction entered the exponential amplification stage after the cycles, suggesting 63 °C as the optimal temperature for the assay.

### 2.4. Specificity of the Proofman-LMTIA

**Figure 4:**
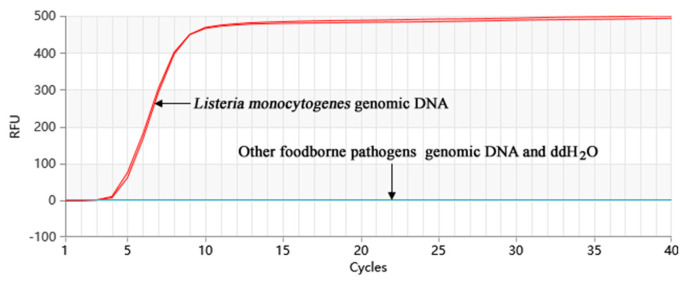
Specificity of the Proofman-LMTIA assay with different DNA types.

The amplification of the genomic DNA from 20 nontarget bacterial strains was performed, and the results are shown in Figure 4. The amplification was just observed in two positive control groups. None of the 20 nontarget bacterial strains showed amplification. Therefore, it can be concluded that the Proofman-LMTIA detection method exhibits good specificity.

### 2.5. The Sensitivity of the Proofman-LMTIA

**Figure 5:**
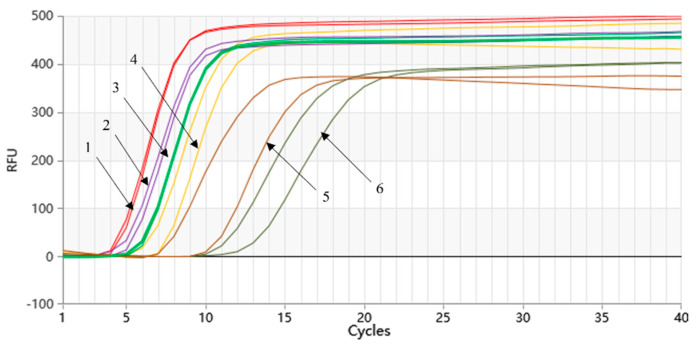
Sensitivity of the Proofman-LMTIA assay with the serial dilution of target DNA. Varying amount of *Listeria monocytogenes* genomic DNA: (1) 1 ng/µL; (2) 100 pg/µL; (3) 10 pg/µL; (4) 1 pg/µL; (5) 100 fg/µL; (6) 10 fg/µL.

The amplification of the gradient-diluted *Listeria monocytogenes* genomic DNA was performed, and the results are shown in Figure 5. The Proofman-LMTIA method was able to detect the DNA of *Listeria monocytogenes* at the lowest concentration (10 fg/µL) in a 40-cycle reaction system. The 20-cycle Proofman-LMTIA system that had a reaction time of 18 min was also tested in the investigation, as shown in Figure 5. Within this timeframe, the LMTIA method was able to detect *Listeria monocytogenes* genomic DNA at a concentration of 100 fg/µL. The slope of the exponential period of the amplification curve is directly proportional to the amplification efficiency, and the larger the slope, the higher the amplification efficiency. When the gene concentration is low, the amplification efficiency is affected, resulting in uneven slope.

### 2.6. Suitability Determination of the Proofman-LMTIA

We conducted simulation testing on frozen fresh pork using the GB 4789.30-2016 method to detect the presence of *Listeria monocytogenes*. The frozen fresh pork was contaminated with a *Listeria monocytogenes* bacterial suspension (10^1^ to 10^8^ CFU/mL) and subjected to plate counting to determine the initial contamination level of *Listeria monocytogenes* in the pork. After 6 h and 12 h of bacterial growth, the genomic DNA of *Listeria monocytogenes* was extracted and used as template for the LMTIA detection. The results are shown in Table 1.

The results showed that after 6 h of bacterial growth in artificially contaminated refrigerated pork, the detection limit of *Listeria monocytogenes* using the established LMTIA method was (6.2 ± 0.5) × 10^1^ CFU/25 g. After 12 h of bacterial growth, the detection limit was found to be 8.0 ± 0.7 CFU/25 g. The performance of the LMTIA method was demonstrated through simulated contaminant experiments, providing theoretical support for further practical sample detection.

### 2.7. Proofman-LMTIA Assay on Retail Food Samples

A total of 30 food samples, including milk, bread, eggs, and meat, were purchased from a Chinese retail market. The results of the Proofman-LMTIA assay for all of these samples were negative, confirming the absence of *L. monocytogenes* in the 30 food samples. The investigation conducted using these food samples provided reliable results.

## 3. Discussion

We used the selected target sequence fragment of *L. monocytogenes* to construct a recombinant positive plasmid, which served as a positive control in the reaction, thereby ensuring the accuracy of our detection results. The nucleic acid sequence with a ladder-type melting temperature curve of *Listeria monocytogenes* was selected using the Oligo 7 software (Molecular Biology Insights, Inc., Cascade, CO, USA) and aligned in GenBank. We selected the target sequence with the aim of optimizing the specificity of the primers. Subsequently, we designed the LMTIA primers probe based on this selected sequence using the online software Primer3Plus (https://dev.primer3plus.com/index.html, accessed on 6 January 2023). We also designed the Proofman probes based on the primer LB sequence, with the fluorophore labeled at the 3′ end mismatch nucleotide and the quencher at the 5′ end nucleotide. This setup was designed to enhance the efficiency and accuracy of the process. They displayed high specificity at 63 °C for *Listeria monocytogenes* (Figure 4). The sensitivity of the method reached 10 fg/µL, which is comparable to or higher than the sensitivity reported for multiple cross displacement amplification (10 fg/µL) [26] and recombinase polymerase amplification (10^3^ cfu/mL) [27].

The method was further evaluated through the analysis of real food samples and artificially contaminated samples, where it demonstrated the suitability and feasibility of the detection. The LMTIA method demonstrated several advantages, including easy operation, high specificity, high sensitivity, and good stability, similar to the loop-mediated isothermal amplification (LAMP) method. Compared to traditional detection methods and PCR, the LMTIA method required less time, minimized false positives, and could complete amplification within 30 min. These characteristics make it suitable for onsite testing and laboratories with limited resources.

The detection of foodborne pathogens has always been a focal point of foodborne disease regulations worldwide. Proofman-LMTIA is a new technology developed by our team based on LMTIA that incorporates the use of Proofman probes. Currently, it is being applied in the detection of foodborne pathogens, food adulteration testing, animal disease detection, edible oil adulteration, and traditional Chinese medicine authentication, among other fields. SYBR Green I is currently the most common fluorescent dye used in PCR. It only binds to the minor groove of double-stranded DNA and does not bind to single-stranded DNA. It remains nonfluorescent when free but emits fluorescence when incorporated into double-stranded DNA. Its greatest advantage is its universality, but its main drawback is the lack of specificity. When there are primer dimers or nonspecific amplification in the PCR reaction, this dye can also bind and emit fluorescence. By replacing SYBR Green I with Proofman probes, specificity can be greatly improved, since the probes only bind to the target fragment and emit fluorescence through proofreading enzyme cleavage. Additionally, the presence of loop primers significantly enhances amplification efficiency, thus reducing the detection time. Moreover, by increasing the number of Proofman probes, multiple detections can be achieved simultaneously.

While the current form of Proofman-LMTIA technology excels in qualitative detection of pathogenic bacteria, it does not provide the quantitative estimation of pathogen content in test samples. Moreover, the method currently lacks the capability to discriminate false positives resulting from nonviable (i.e., dead) bacterial cells. Therefore, the next step would be to combine LMTIA technology with other techniques to achieve quantitative detection, qualitative detection of multiple targets, and point mutation detection. The development of new methods could potentially broaden the application of this technology in food safety testing, animal disease detection, and molecular diagnostics.

## 4. Materials and Methods

### 4.1. Construction of the Positive Plasmid

#### 4.1.1. Bacterial Cultivation

*Listeria monocytogenes* BNCC 185986 was streaked on TSA agar plates (purchased from Qingdao Hope Bio-Technology Co., Ltd., Qingdao, China) and incubated at 37 °C for 12–16 h. A single colony from the TSA agar culture was picked using a sterile loop inoculated in 10 mL of LB broth and incubated overnight at 37 °C with shaking at 200 rpm.

#### 4.1.2. Bacterial DNA Extraction

One milliliter of the fresh overnight bacterial culture in LB broth was collected for genomic DNA extraction. The extraction was performed using the Bacterial Genomic DNA Extraction Kit from Solarbio according to the manufacturer’s instructions. The concentration of extracted DNA was determined using the NanoDrop One Microvolume UV-Vis Spectrophotometer.

#### 4.1.3. PCR Primer

By aligning the *Listeria monocytogenes* ATCC 51779 (CP025567.1) genome (obtained from the GenBank database) with our strain, the PCR primers targeting the specific region where the melting temperature curve of the sequence is of a ladder type were designed using Oligo 6.0 software. The reverse primer was used for sequencing. Primers were synthesized by Universal Biotech (Anhui) Co., Ltd., Chuzhou, China. Sequences of the PCR primers is shown in Table 2.

#### 4.1.4. PCR Reaction

The PCR amplification was performed using a 25 μL total reaction volume, with the extracted genomic DNA of *Listeria monocytogenes* as the template. The reaction mixture and conditions are outlined in Table 3. The amplification program consisted of 30 cycles with the following program: denaturation at 98 °C for 5 s, annealing at 57 °C for 10 s, and extension at 72 °C for 5 s. The PCR amplicons were subjected to 1% agarose gel electrophoresis for visualization.

#### 4.1.5. Sequence Examination of the PCR Target

The PCR amplicons obtained from the gel electrophoresis, as described in Section 4.1.4, were purified using the SanPrep column DNA gel recovery kit from Shanghai Sangon Biotech Co., Ltd., Shanghai, China. The concentration of the PCR amplicons was quantified using the NanoDrop One microvolume nucleic acid and protein analyzer and then stored at −20 °C. The purified PCR products were ligated into the cloning vector pMD-19T using greater than 75 ng/μL DNA. The ligated plasmid was heat-shocked into DH5α competent cells and plated on agar plates for incubation at 37 °C for 12–16 h. The RNase and proteinase k dissolved in TE were used in enzymatic digestion. Single colonies were selected and inoculated into LB liquid medium containing ampicillin and incubated at 37 °C with shaking at 200 rpm for 12–16 h. A 2 mL aliquot of the bacterial culture was used for plasmid extraction, and the size of the inserted fragments was confirmed by enzymatic digestion. The DNA target with the expected length was sent to Universal Biotech (Anhui) Co., Ltd., for sequencing.

### 4.2. Target Sequence and Its Primers for LMTIA

**Figure 6:**
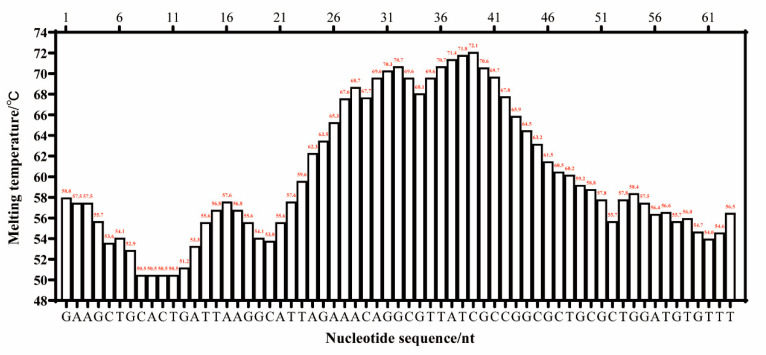
The melting temperature curve of the target sequence for the detection of *Listeria monocytogenes* using the Proofman-LMTIA method.

There were three criteria for the target sequence selection, as described by Wang et al. [18]: the melting temperature curve of the sequence was of a ladder type; the GC content of the sequence was generally 40–80%; and the sequence had high specificity. The whole genome of *L. monocytogenes* ATCC 51779 (CP025567.1) was downloaded from the GenBank gene database. Using Oligo 7.0 software, the sequences were selected based on their melting temperature profiles. These sequences were then analyzed using BLAST to identify highly specific target sequences. The target length for the LMTIA primers was determined to be 83 bp, and the melting temperature curve displayed a gradient pattern, as shown in Figure 6. Its GC content was 51.81%, and the sequence was highly specific to the species of *L. monocytogenes*. Therefore, this sequence was selected as the target sequence for the LMTIA primer design. The selected target sequence was used to design LMTIA primers and Proofman probes using the online primer design software Primer 3 Plus (http://www.primer3plus.com, accessed on 6 January 2023). The specific sequences can be found in Table 4. The same or complementary fragments of the primer and target sequences were labeled with the same color.

### 4.3. Optimization of the Proofman-LMTIA Reaction Temperature

For each reaction, 10 ng/µL of the recombinant plasmid served as the positive control, while ddH_2_O served as the negative control. Both the positive and negative controls were added at a volume of 2 μL. Two replicates were set up for each control. The temperature gradient was set using the Gentier 96E fully automated medical PCR analysis system, with temperatures ranging from 59 °C to 64 °C. Fluorescence signals were collected every 90 s for a total of 40 cycles. We chose the temperature with the highest amplification efficiency as the optimal amplification temperature.

### 4.4. Specificity Determination 

Target strains and nontarget strains (Table 5) from the laboratory collection were cultured overnight in appropriate media. Genomic DNA was extracted using the bacterial genome DNA extraction kit from Beijing Solarbio Company. The optimized reaction temperatures obtained in Section 4.3 were used to test the extracted DNA from different bacterial species to validate the specificity of the primers.

### 4.5. Sensitivity Determination

The genomic DNA of *Listeria monocytogenes* was serially diluted to a final concentration of 10 ng/μL, 1 ng/μL, 100 pg/μL, 10 pg/μL,1 pg/μL, 100 fg/μL, 10 fg/μL, and 1 fg/μL for evaluating the sensitivity of the Proofman-LMTIA.

### 4.6. Suitability Determination

Fresh pork purchased from a retail store was chosen as a simulated sample, which was cut into pieces and washed with physiological saline to remove surface contaminants, dried on a clean bench, and exposed to UV light for 20 min. According to the GB 4789.30-2016 method, no *Listeria monocytogenes* were detected in the used pork samples. Aseptically minced pork samples (25 g each) were mixed with 75 mL of sterile water in sterile bottles. The pork samples were prepared by individually adding 1 mL of different diluents of *Listeria monocytogenes* from 10^1^ to 10^8^ CFU/mL. The inoculated samples were incubated at room temperature for 20 min. The cell counts of the inoculated *Listeria monocytogenes* were confirmed by plating. Also, the inoculated samples were incubated at 37 °C, and the samples were taken for examination at 6 and 12 h. For all of the samples, the genomic DNA of *Listeria monocytogenes* was extracted, and the detection of *Listeria monocytogenes* was performed using the optimized Proofman-LMTIA method.

### 4.7. Detection of Actual Food Samples

Thirty food samples, including milk, bread, eggs, pork, beef, and lamb, were procured from the market and subjected to testing using both the Proofman-LMTIA method and a Lakicevic B [28]-based PCR method. The results were further validated in accordance with the GB 4789.30-2016 national standard for the microbiological examination of *Listeria monocytogenes*. This validation procedure encompasses sampling, enrichment, plate culture, and identification.

## 5. Conclusions

The LMTIA primers were specifically designed to target specific genes of *L. monocytogenes*, characterized by a ladder-type melting temperature curve. The resulting melting temperature curve exhibited a ladder pattern, indicating successful primer design. Utilizing this design, the Proofman-LMTIA assay was successfully developed and implemented for the detection of *L. monocytogenes* in both artificially contaminated and actual food samples. The LMTIA primers demonstrated excellent specificity at the optimized temperature of 63 °C, with no cross-reaction observed when distinguishing *L. monocytogenes* DNA from other foodborne pathogen DNAs, including Escherichia coli, Staphylococcus aureus, and Salmonella. The primers proved effective in detecting an initial inoculation level of 8.0 ± 0.7 CFU/25 g in pork samples after 12 h of cultivation. This detection level surpasses that of the plate culture method and is comparable to other molecular detection methods.

To further validate the efficacy of the method, 30 food samples purchased from the market were tested for the presence of *L. monocytogenes*, resulting in a 100% successful detection rate. These findings underscore the rapidity, simplicity, and cost-effectiveness of the Proofman-LMTIA assay, which holds significant potential for the identification of foodborne pathogens in food. Its implementation can contribute to enhanced food safety monitoring and the protection of consumer rights.

## Figures and Tables

**Table 1:** Sensitivity of the Proofman-LMTIA for detecting *Listeria monocytogenes* in artificially contaminated pork.

Sample No.	Cell Counts (CFU/25 g)	Proofman-LMTIA	
		6 h	12 h
1	(3.5 ± 0.3) × 10^7^	+	+
2	(6.4 ± 1.8) × 10^6^	+	+
3	(4.6 ± 2.0) × 10^5^	+	+
4	(3.2 ± 0.8) × 10^4^	+	+
5	(6.5 ± 0.4) × 10^3^	+	+
6	(3.3 ± 1.2) × 10^2^	+	+
7	(6.2 ± 0.5) × 10^1^	+	+
8	8.0 ± 0.7	−	+

“+” Indicated as a positive result; “−” indicated as a negative result.

**Table 2:** Sequences of the PCR primers.

Primer	Length of the DNA Target
F: 5′-TTGATGTGGGTGTTGGAGGC-3′	880 bp
R: 5′-CACCTGGAAGTAAATCAGCAACC-3′	

**Table 3:** Reaction system of the PCR.

Component	Volume
PCR Mix	12.5 µL
Forward primer (10 µM)	1 µL
Reverse primer (10 µM)	1 µL
DNA template	2 µL
ddH_2_O	8.5 µL

**Table 4:** Primers for the LMTIA and the target sequence.

Primer	Sequence (5′–3′)
F (Forward primer)	GCGCCGGCGATATTTTGAAGCTGCACTGATTAAGGC
B (Reverse primer)	TATCGCCGGCGCTTTTCCAATCTTAGGCTCGAACTCA
LF (Loop primer forward)	ACGCCTGTTTCTAAT
LB (Loop primer reverse)	TGCGCTGGATGTGTT
Proofman probe	ACGCCTGTTTCTAAC
Target sequence	
GAAGCTGCACTGATTAAGGC ATTAGAAACAGGCGT TATCGCCGGCGC TGCGCTGGATGTGTT TGAGTTCGAGCCTAAGATTGG	

**Table 5:** Experimental strains for specific detection.

No.	Bacteria	Strain
1	*Listeria monocytogenes*	BNCC 185986
2	*Staphylococcus aureus*	ATCC 25923
3	*Staphylococcus aureus*	ATCC 6538
4	*Escherichia coli*	ATCC 25922
5	*Escherichia coli* O157:H7	NCTC 12900
6	*Salmonella* spp.	ATCC 13076
7	*Listeria seeligeri*	ATCC 35967
8	*Listeria ivanovii*	ATCC 19119
9	*Shigella boydii*	CMCC(B) 51522
10	*Shigella dysenteriae*	CMCC(B) 51105
11	*Shigella flexneri*	CMCC(B) 51572
12	*Shigella sonnei*	ATCC 25931
13	*Vibrio parahaemolyticus*	ATCC 17082
14	*Bifidobacterium bifidum*	CICC 6071
15	*Bifidobacterium breve*	CICC 6079
16	*Bifidobacterium longum*	CICC 6068
17	*Bifidobacterium adolescentis*	CICC 6070
18	*Bifidobacterium infantisreuter*	BNCC 341709
19	*Bifidobacterium animalis*	CICC 6165
20	*Bifidobacterium dentium*	BNCC 360738

## Data Availability

The data presented in this study are available upon request from the corresponding author.

## Sample Availability

Samples of the compounds are not available from the authors.
